# Synthesis, Spectral Characterization and Analgesic Activity of 2-Methylthio-1,4-Dihydropyrimidines

**Published:** 2011

**Authors:** Ramesh Sawant, Varsha Sarode

**Affiliations:** *P.D.V.V.P.F’s College of Pharmacy, ViladGhat, Ahmednagar, 414111, India.*

**Keywords:** 2-Methylthio-1, 4-dihydropyrimidine, Biginelli reaction, Synthesis; Analgesic

## Abstract

A series of 2-methylthio-1,4-dihydropyrimidine derivatives (II) were synthesized in good yields by alkylation of 1,2,3,4-tetrahydropyrimidines (I) with methyl iodide in the presence of pyridine. Their structures were confirmed by elemental analysis, IR and ^1^H NMR spectra. The compounds were tested for analgesic activity by acetic acid induced writhing method. Compounds IIh, IIe, IIk and IIl showed excellent to good analgesic activity. Other compounds showed moderate analgesic activity. The observed analgesic activity is mainly because of inhibition of the peripheral pain mechanism by the title compounds.

## Introduction

Multicomponent reactions (MCRs) are of increasing importance in organic and medicinal chemistry. Multicomponent condensations involve three or more compounds reacting in a single event to form a product, which contains the essential parts of all the starting materials. Among the wide range of heterocycles explored to develop pharmaceutically important molecules, pyrimidine has played an important role in medicinal chemistry. Dihydropyrimidines are being looked as an important class of molecules since many of them are clinical candidates. In recent years acid-catalyzed cyclocondensation of acetoacetate with aldehydes and (thio) ureas, known as the Biginelli reaction, has attracted significant attention (1-9). The resulting dihydropyrimidines (DHPMs) have been reported to have antibacterial (10), antiviral (11), anti-inflammatory (12), analgesic (13), antihypertensive as well as calcium channel blocker (14, 15) and antioxidant (16) activities. Recently, structurally simple DHPM derivative monastrol has emerged as a mitotic kinesin Eg5 motor protein inhibitor for the development of anticancer drugs (17). Furthermore, the biological activity of several recently isolated marine alkaloids has also been attributed to the dihydropyrimidinone moiety in the structure. Among them the batzelladine alkaloids A and B which inhibit the binding of HIV envelope protein gp-120 to human CD4 cells are potential compounds in AIDS therapy (18).

Pain is defined as neuralgia, an unpleasant sensory experience associated with tissue damage, such as injury, inflammation or cancer, but severe pain can arise independently of any obvious predisposing cause, or persist long after the precipitating injury has healed. Acetic acid induced writhing model represents pain sensation by triggering localized inflammatory response. Such pain stimulus leads to the release of free arachidonic acid from tissue phospholipids (19). The abdominal constriction response induced by acetic acid is a sensitive procedure to establish peripherally acting analgesics. The response is thought to be mediated by peritoneal mast cells (20), acid sensing ion channels (21) and the prostaglandin pathways (22). Non-steroidal anti-inflammatory drugs (NSAIDs) are among the most widely used therapeutics, primarily for the treatment of pain and inflammation. However, long-term clinical usage of NSAIDs is associated with significant side effects of gastrointestinal lesions, bleeding, and nephrotoxicity (23). 

Pyrimidine and condensed pyrimidine derivatives possessing anti-inflammatory and analgesic activities are well documented in the literature (24). In the present work some novel analgesic 2-methylthio-1, 4-dihydropyrimidines are synthesized and structurally characterized.

## Experimental

Melting points were determined in open capillaries and are uncorrected. All compounds were characterized by elemental analysis, IR and ^1^H NMR spectra. The IR spectra were recorded on a JASCO FT-IR 4100 spectrometer, using KBr discs. The ^1^H NMR spectra were obtained on a Varian-NMR-mercury300 spectrometer in DMSO-d6 as solvent and TMS as internal standard, chemical shifts are given in ppm.

*General procedure for the synthesis of compounds (IIa-IIl)*

A mixture of appropriate aldehyde (0.02 mole), acetoacetate (0.02 mole), thiourea (0.03 mole), catalyst aluminium chloride (0.01 mole) in methanol (10 mL) and concentrated hydrochoric acid (2 drops) was placed in round bottom flask. The mixture was stirred well and then refluxed. The completion of reaction was monitored by thin layer chromatography. After cooling, precipitate was formed which was filtered and washed with cold methanol (I).

Compound I (0.01 mole), methyl iodide (0.011 mole) in methanol (20 mL) was placed in round bottom flask and refluxed for 2 h. Pyridine (0.037 mole) was then added and refluxed again for 10 min After cooling, the reaction mixture was poured onto crushed ice (200 g) and stirred for 5 min. Compound II obtained was filtered.

*Ethyl 6-methyl-2-(methylthio)-4-phenyl-1,4-dihydropyrimidine-5-carboxylate (IIa)*

Yield: 82.10 %; m.p. 160-162ºC; IR-(KBr) cm^-1^: 3318.89 (NH), 2371.05 (S-CH_3_), 1659.45 (C=O), 1573.63 (C=N); ^1^H NMR (300 MHz, DMSO): 14.18 (s, 1H, NH), 7.75-7.20 (m, 5H, C_6_H_5_), 5.988 (S, 1H, CH), 3.98 (q, 2H, OCH_2_CH_3_ ), 2.35 (s, 1H, S-CH_3_), 2.193 (s, 3H, CH_3_), 1.11 (t, 3H, OCH_2_CH_3_).

*Methyl 6-methyl-2-(methylthio)-4-phenyl-1,4-dihydropyrimidine-5-carboxylate (IIb)*

yield: 67.51 %; m.p. 100-102ºC; IR-(KBr) cm^-1^: 3316.96 (NH), 2362.37 (S-CH_3_), 1708.62 (C=O), 1646.91 (C=N); ^1^H NMR (300 MHz, DMSO): 14.876 (s, 1H, NH), 7.751-7.201 (m, 5H, C_6_H_5_), 5.988 (s, 1H, CH), 3.71 (s, 3H, OCH_3_), 2.351 (s, 1H, S-CH_3_), 2.193 (s, 3H, CH_3_).

*Ethyl 4-(4-methoxyphenyl)-6-methyl-2-(methylthio)-1,4-dihydropyrimidine-5-carboxylate (IIc)*

yield: 78.94 %; m.p. 116-117ºC; IR-(KBr) cm^-1^: 3318.89 (NH), 2345.98 (S-CH_3_), 1649.8 (C=0), 1150.33(C-O); ^1^H NMR (300 MHz, DMSO): 14.876 (s, 1H, NH), 7.68-7.069 (m, 4H, C_6_H_4_), 5.988 (s, 1H, CH), 3.98 (q, 2H, OCH_2_CH_3_), 3.605 (s, 3H, OCH_3_), 2.351 (s, 3H, S-CH_3_), 2.193 (s, 3H, CH_3_), 1.11 (t, 3H, OCH_2_CH_3_).

*Methyl 4-(4-methoxyphenyl)-6-methyl-2-(methylthio)-1,4-dihydropyrimidine-5-carboxylate (IId)*

yield: 73.68 %; m.p. 146-147ºC; IR-(KBr) cm^-1^: 3318.89 (NH), 2364.3 (S-CH_3_), 1654.62 (C=O), 1249.65 (C-O); ^1^H NMR (300 MHz, DMSO):14.87 (s, 1H, NH), 7.68-7.069 (m, 4H, C_6_H_4_), 5.988 (s, 1H, CH), 3.708 (s, 3H, OCH_3_), 3.605 (s, 3H, OCH_3_), 2.351 (s, 3H, S-CH_3_), 2.193 (s, 3H, CH_3_).

*Ethyl 4-(4-chlorophenyl)-6-methyl-2-(methylthio)-1,4-dihydropyrimidine-5-carboxylate (IIe)*

yield: 92.98 %; m.p. 138-139ºC; IR-(KBr) cm^-1^: 3341.07 (NH), 2358.52 (S-CH_3_), 1674.87 (C=O), 1574.59 (C=N), 745.35 (C-Cl); ^1^H NMR (300 MHz, DMSO): 14.876 (s, 1H, NH), 7.778-7.396 (m, 4H, C_6_H_4_), 5.988 (s, 1H, CH), 3.98 (q, 2H, OCH_2_CH_3_), 2.351 (s, 3H, S-CH_3_), 2.193 (s, 3H, CH_3_), 1.11 (t, 3H, OCH_2_CH_3_).

*Methyl 4-(4-chlorophenyl)-6-methyl-2-(methylthio)-1,4-dihydropyrimidine-5-carboxylate (IIf)*

yield: 57.89 %; m.p. 130-132ºC; IR-(KBr) cm^-1^: 3308.29 (NH), 2372.01 (S-CH_3_), 1659.45 (C=O), 1561.09 (C=N), 782.95 (C-Cl); ^1^H NMR (300 MHz, DMSO):14.876 (s, 1H, NH), 7.778-7.396 (m, 4H, C_6_H_4_), 5.988 (s, 1H, CH), 3.708 (s, 3H, OCH_3_), 2.351 (s, 3H, S-CH_3_), 2.193 (s, 3H, CH_3_).

*Ethyl 4-[4-(dimethylamino)phenyl]-6-methyl-2-(methylthio)-1,4-dihydropyrimidine-5-carboxylate (IIg)*

yield: 62.93 %; m.p.124-126ºC; IR-(KBr) cm^-1 ^3334.32 (NH), 2371.05 (S-CH_3_), 1654.62 (C=O), 1514.81 (C=N); ^1^H NMR (300 MHz, DMSO):14.876 (s, 1H, NH), 7.532-6.577 (m, 4H, C_6_H_4_), 5.988 (s, 1H, CH), 3.98 (q, 2H, OCH_2_CH_3_), 2.831 (s, 6H, N(CH_3_)_2_), 2.351 (s, 3H, S-CH_3_), 2.193 (s, 3H, CH_3_), 1.11 (t, 3H, OCH_2_CH_3_),.

*Methyl 4-[4-(dimethylamino)phenyl]-6-methyl-2-(methylthio)-1,4-dihydropyrimidine-5-carboxylate (IIh)*

yield: 75.75 %; m.p. 214-216ºC; IR-(KBr) cm^-1^: 3327.57 (NH), 2358.52 (S-CH_3_), 1668.12 (C=O), 1555.31 (C=N); ^1^H NMR (300 MHz,DMSO): 14.876 (s, 1H, NH), 7.532-6.577 (m, 4H, C_6_H_4_), 5.988 (s, 1H, CH), 3.708(s, 3H, OCH_3_), 2.831 (s, 6H, N(CH_3_) _2_), 2.351 (s, 3H, S-CH_3_), 2.193 (s, 3H, CH_3_),.

*Ethyl 6-methyl-2-(methylthio)-4-(4-nitrophenyl)-1,4-dihydropyrimidine-5-carboxylate (IIi)*

yield: 83.68 %; m.p.180-182ºC; IR-(KBr) cm^-1^: 3235.97 (NH), 2372.01 (S-CH_3_), 1693.19 (C=O), 1528.31 (Asy Ar-NO_2_), 1349 (Sym Ar-NO_2_), 855.27 (C-N); ^1^H NMR (300 MHz, DMSO): 14.876 (s, 1H, NH), 7.89-8.10 (m, 4H, C_6_H_4_), 5.988 (s, 1H, CH), 3.98 (q, 2H, OCH_2_CH_3_), 2.351 (s, 3H, S-CH_3_), 2.193 (s, 3H, CH_3_), 1.11 (t, 3H, OCH_2_CH_3_).

*Ethyl 6-methyl-2-(methylthio)-4-(3,4,5-trimethoxyphenyl)-1,4-dihydropyrimidine-5-carboxylate (IIj)*

yield: 75.14 %; m.p.206-208ºC; IR-(KBr)cm^-1^: 3288.04 (NH), 2345.02 (S-CH_3_), 1669.09 (C=O), 1573.63 (C=N),1184, 1123 (C-O); ^1^H NMR (300 MHz, DMSO): 14.87 (s, 1H, NH), 5.874 (s, 1H, CH), 3.98 (q, 2H, OCH_2_CH_3_), 3.83 (s, 9H, OCH_3_), 2.351 (s, 3H, S-CH_3_), 2.193 (s, 3H, CH_3_), 1.11 (t, 3H, OCH_2_CH_3_).

*Ethyl 6-methyl-2-(methylthio)-1,4-dihydropyrimidine-5-carboxylate (IIk)*

yield: 65.47 %; m.p. 210-211ºC; IR-(KBr) cm^-1^: 3209.93 (NH), 2352.73 (S-CH_3_), 1659.45 (C=O), 1501.31 (C=N); ^1^H NMR (300 MHz, DMSO): 15.179 (s, 1H, NH), 4.723 (S, 2H, CH_2_), 4.089 (q, 2H, OCH_2_CH_3_), 2.402 (s, 3H, S-CH_3_), 2.173 (s, 3H, CH_3_), 1.222 (t, 3H, OCH_2_CH_3_).

*Methyl 6-methyl-2-(methylthio)-1,4-dihydropyrimidine-5-carboxylate (IIl)*

yield: 75.14 %; m.p. 198-200ºC; IR-KBr (cm^-1^): 3318.07 (NH), 2358.52 (S-CH_3_), 1668.12 (C=O), 1594.84 (C=N); ^1^H NMR (300 MHz, DMSO): 15.17 (s, 1H, NH), 4.723 (S, 2H, CH_2_), 3.705 (s, 3H, OCH_3_), 2.402 (s, 3H, S-CH_3_), 2.173 (s, 3H, CH_3_).

*Analgesic activity*

*Experimental animals*

Swiss albino mice of either sex weighing 25 to 30 g maintained in our college animal house were used for the study. The animals were divided into fourteen groups each containing six mice. Experiments reported in this study were carried out in accordance with local guidelines for the care of laboratory animals of PDVVPF’s Medical College, Ahmednagar. 

*Writhing test method*

Analgesic activity was carried out by acetic acid induced writhing method in Swiss albino mice (25-30 g). 0.1 mL of a 0.6 % aqueous acetic acid solution was injected intraperitoneally (IP) as writhing inducing agent. In each group six mice were kept. Mice were kept individually in test cage, before acetic acid injection. Screening of analgesic activity was performed after oral administration of test compounds at a dose of 50 mg/kg. All compounds were dissolved in sterile water for injection (SWF). Diclofenac was used as reference drug. After 1 h of drug administration 0.10 mL of 0.6 % acetic acid solution was given to mice intraperitoneally. Stretching movements consisting of arching of the back, elongation of body and extension of hind limbs were counted for 10 min of acetic acid injection. The analgesic activity was expressed in terms of percentage inhibition. Percentage analgesic activity was calculated as follows:

Analgesic activity (%inhibition) = (n-n’ / n) ×100

Where, n = Mean number of writhes of control group.

n’ = Mean number of writhes of test group.

*Statistical analysis*

Values are expressed as mean ± SEM and data was analyzed by ANOVA followed by Dunnet’s test. p< 0.01 was considered as significant.

## Results and Discussion

**Figure 1:**
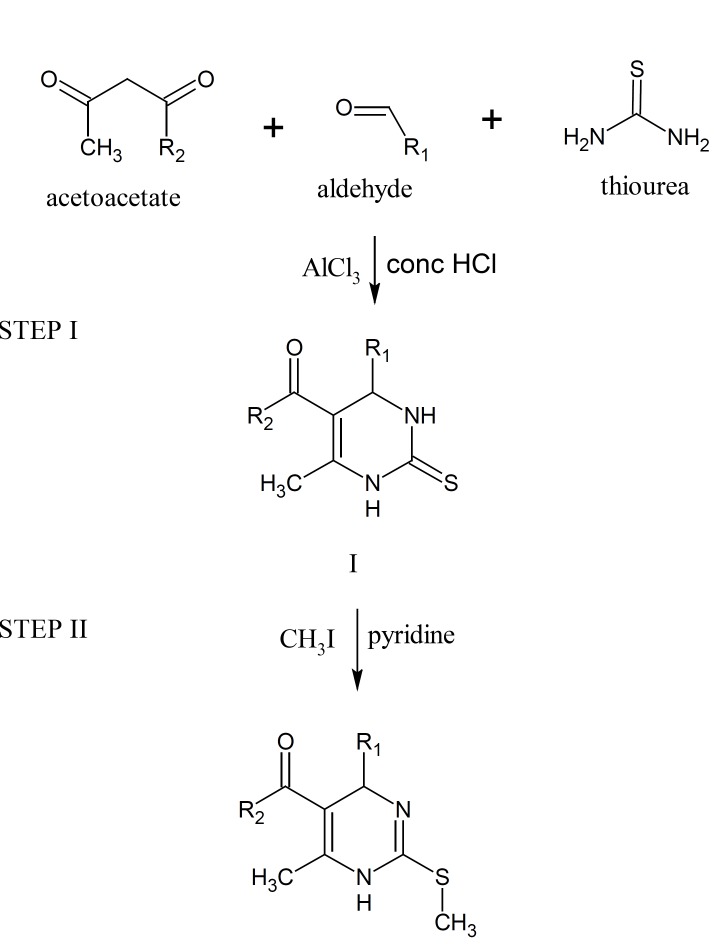
Synthesis of 2-methylthio-1,4-dihydropyrimidines

The 1,2,3,4-tetrahydropyrimidine-2-thione derivatives were prepared by Biginelli three-component reaction of appropriate aldehydes, acetoacetate and thiourea. Alkylation reaction was used for the formation of C-2 modified DHPM derivatives of type II. The target molecules, 2-methylthio-1,4-dihydropyrimidine derivatives were synthesized from the respective tetrahydropyrimidine-2-thiones by reaction with methyl iodide in the presence of pyridine (Figure 1, Table 1).

**Table 1:** Chemical structures and physicochemical characteristic of title compound (IIa-IIl).

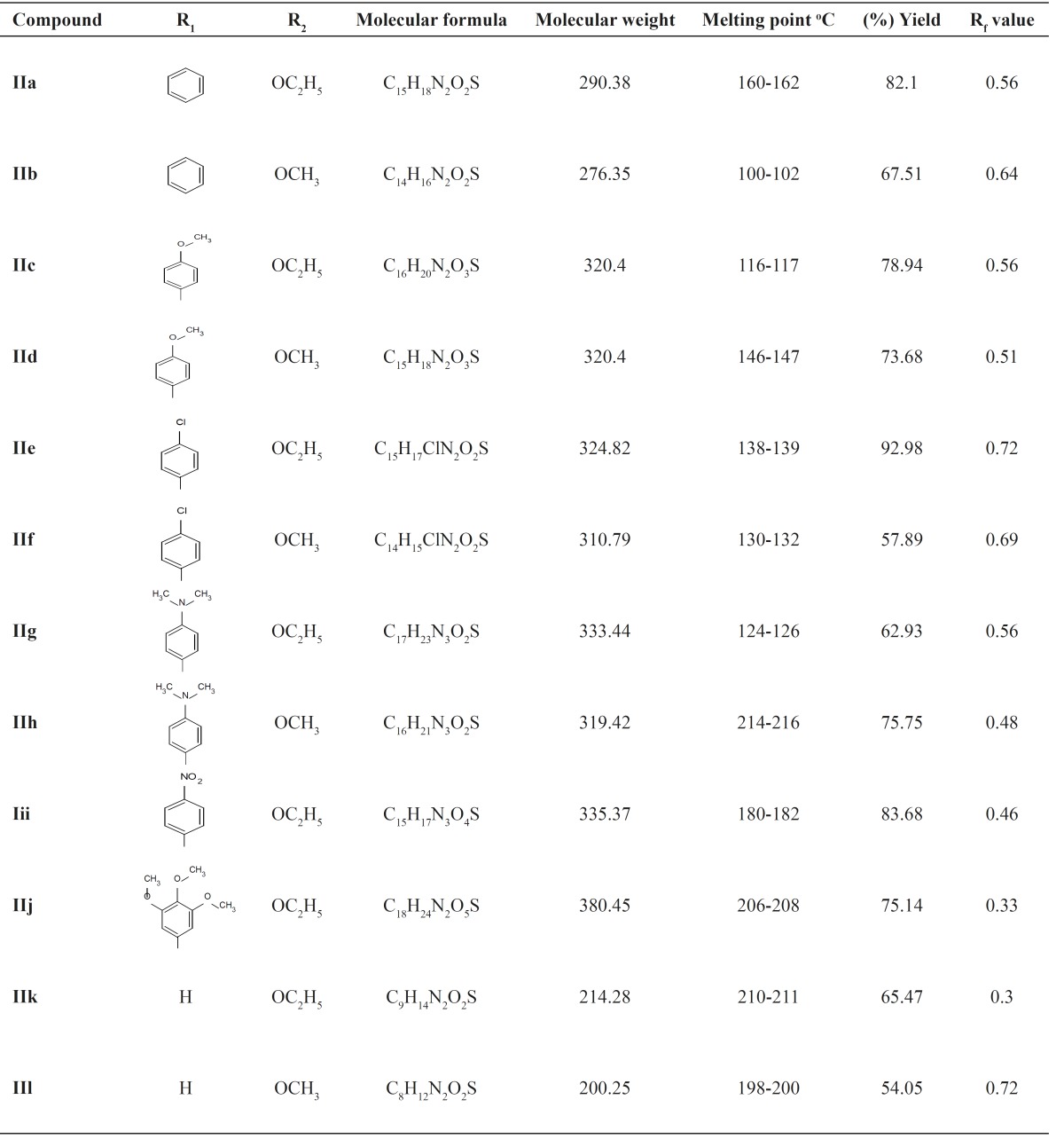

The purity of the compounds was checked by thin layer chromatography. Their structures were confirmed by elemental analysis, IR and ^1^H NMR spectra. The amount of carbon, hydrogen and nitrogen found by elemental analysis is in good agreement with calculated. 

**Figure 2:**
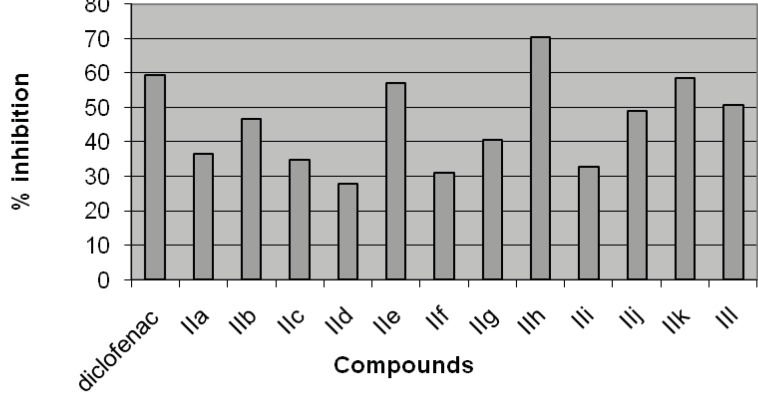
Effect of compounds (IIa-IIl) on acetic acid induced writhing in mice

The analgesic activity of the synthesized compound (IIa-IIl) was evaluated by acetic acid induced writhing test (Table 2, Figure 2). The compound IIh bearing p-dimethylaminophenyl substituent at the fourth position of 1, 4-dihydropyrimidine exhibited maximum analgesic activity (70.32%). Whereas compounds IIk and IIl with unsubstituted fourth position of 1,4-dihydropyrimidine showed good analgesic activity (58.45 and 50.68 %). If p-chlorophenyl group is placed at the fourth position of 1,4-dihydropyrimidine compound IIe again showed good analgesic activity (57.08 %). The compound IIf with p-chlorophenyl group at the fourth position and methyl ester at fifth position of 1,4-dihydropyrimidine showed lowest analgesic activity in the present series.

**Table 2:** Analgesic activity by acetic acid induced withing method of compound (IIa-IIl) at 50 mg/kg dose.

**Compound**	**No of writhing: mean±SEM**	**% Inhibition**
Control	73 ± 1.00	-
Diclofenac	29.66 ± 0.33	59.36
IIa	46.33 ± 0.33**	36.53
IIb	39 ± 0.577**	46.57
IIc	47.66 ± 1.45**	34.71
IId	52.66 ± 0.33**	27.86
IIe	31.33 ± 1.85**	57.08
IIf	50.33 ± 0.88**	31.05
IIg	43.33 ± 0.88**	40.50
IIh	21.66 ± 0.88**	70.32
IIi	49 ± 1.15**	32.87
IIj	37.33 ± 0.88**	48.86
IIk	30.33 ± 0.66**	58.45
IIl	36 ± 0.57**	50.68

**p < 0.01 was considered significant

Chikhale*et al. *(13) have reported that ethyl 6-methyl-2-methoxy-3-(1-phenylethanone)-4-(4-methoxyphenyl)-1,2,3,4-tetrahydropyrimidine-5-carboxylate containing methoxy group at fourth position of 1,2,3,4-tetrahydropyrimidine has analgesic activity comparable with the standard drug ibuprofen The SAR study suggest that the phenyl ring, at the fourth position of 1,4-dihydropyrimidines, substituted with electron donating hydrophobic group is required for the analgesic activity. 

## Conclusion

Various 2-methylthio-1,4-dihydropyrimidines are synthesized in reasonable yield by a simple, efficient and one-pot reaction. Compounds IIa-IIl exhibits significant analgesic activity in acetic acid induced writhing test at a dose of 50 mg/kg. The analgesic activity was found to be significant on acetic acid induced writhing model (p < 0.01) and thus it appears that the test compounds inhibits predominantly the peripheral pain mechanism.
